# Diagnosis, Classification, and Assessment of the Underlying Etiology of Uveitis by Artificial Intelligence: A Systematic Review

**DOI:** 10.3390/jcm12113746

**Published:** 2023-05-29

**Authors:** Robin Jacquot, Pascal Sève, Timothy L. Jackson, Tao Wang, Antoine Duclos, Dinu Stanescu-Segall

**Affiliations:** 1 Department of Internal Medicine, Croix-Rousse Hospital, Hospices Civils de Lyon, Claude Bernard-Lyon 1 University, F-69004 Lyon, France; pascal.seve@chu-lyon.fr; 2 Research on Healthcare Performance (RESHAPE), INSERM U1290, Claude Bernard Lyon 1 University, F-69000 Lyon, France; antoine.duclos@chu-lyon.fr; 3 Department of Ophthalmology, King’s College Hospital, London SE5 9RS, UK; t.jackson1@nhs.net; 4 Faculty of Life Science and Medicine, King’s College London, London SE5 9RS, UK; 5 DISP UR4570, Jean Monnet Saint-Etienne University, F-42300 Roanne, France; tao.wang@univ-st-etienne.fr; 6 Department of Ophthalmology, La Pitié-Salpêtrière Hospital, APHP, F-75013 Paris, France; dinustanescusegall@gmail.com

**Keywords:** uveitis, diagnosis, etiology, decision support system, artificial intelligence, Bayes’ theorem, algorithms

## Abstract

Recent years have seen the emergence and application of artificial intelligence (AI) in diagnostic decision support systems. There are approximately 80 etiologies that can underly uveitis, some very rare, and AI may lend itself to their detection. This synthesis of the literature selected articles that focused on the use of AI in determining the diagnosis, classification, and underlying etiology of uveitis. The AI-based systems demonstrated relatively good performance, with a classification accuracy of 93–99% and a sensitivity of at least 80% for identifying the two most probable etiologies underlying uveitis. However, there were limitations to the evidence. Firstly, most data were collected retrospectively with missing data. Secondly, ophthalmic, demographic, clinical, and ancillary tests were not reliably integrated into the algorithms’ dataset. Thirdly, patient numbers were small, which is problematic when aiming to discriminate rare and complex diagnoses. In conclusion, the data indicate that AI has potential as a diagnostic decision support system, but clinical applicability is not yet established. Future studies and technologies need to incorporate more comprehensive clinical data and larger patient populations. In time, these should improve AI-based diagnostic tools and help clinicians diagnose, classify, and manage patients with uveitis.

## 1. Introduction

Uveitis is an intraocular inflammation with a reported incidence of 17 to 52 per 100,000 people and a prevalence of 38–284 per 100,000 [1]. It is the fifth leading cause of blindness worldwide, with vision loss most often mediated by macular edema, glaucoma, and retinal ischemia [2]. In 2021, the Standardization of Uveitis Nomenclature (SUN) system set out classification criteria for uveitis [3] based on anatomic location, onset, duration, course, and severity.

Uveitis is associated with approximately 80 different etiologies, the most common of which are infectious, autoimmune, or autoinflammatory [4]. The causal epidemiology can vary from country to country. For example, in some countries, genetic factors such as HLA-B27 are key, while in others, diseases such as sarcoidosis or infections such as tuberculosis may be over-represented. Hence, the epidemiological background of patients with uveitis is important in determining the underlying cause. In addition, ocular (anatomic location, laterality, chronicity, and associated signs) and extraocular (clinical examination and systemic workup) features may help orient the diagnosis. The plethora of potentially relevant variables makes the etiological diagnosis complex [4,5]. In Western countries, one-quarter of uveitis diagnoses are related to an isolated ophthalmic disease, one-quarter to a confirmed systemic disease, one-quarter to a suspected systemic disease, and in between 23% and 44% of cases, the cause remains undetermined, varying by center [6].

A small number of studies have focused on the best diagnostic approach for a given type of uveitis. Only one was a controlled study, comparing a “standardized 3-step approach” to an “open” strategy for investigation, with allocation based on simple ocular characteristics, namely the location and type of uveitis [7].

Because of these diagnostic complexities and a lack of strong evidence or consensus to guide diagnostic approaches, inexperienced or non-specialist clinicians may find uveitis a challenging condition to diagnose and classify. In recent years, machine learning and deep learning have shown increasing utility in the analysis, interpretation, and exploitation of mass data. In medicine, Bayesian belief networks have been proposed as a tool to assist in the differential diagnosis of medical conditions [8,9]. More recently, researchers applied this technique to the diagnosis of uveitis, with promising results [10,11]. Ophthalmological AI algorithms have been applied mainly in diabetic retinopathy screening, age-related macular degeneration, and corneal disease [12]. Most of these algorithms could be applied for uveitis ophthalmological exams. It would help to have a synthesis of the available literature and identify what further evidence is required.

This article aims to synthesize the literature on the use of AI in the diagnosis of uveitis, its classification, and its assistance in determining the underlying cause.

## 2. Materials and Methods

### 2.1. Search Strategy and Selection Criteria

A systematic literature review was completed on 6 March 2023. The review followed the Preferred Reporting Items for Systematic Reviews and Meta-Analyses (PRISMA) guidelines, and the protocol was registered in the International Prospective Register of Ongoing Systematic Reviews (PROSPERO) (ID: 409568) [13]. The search strategy was conducted across four electronic databases (PubMed, Cochrane, MEDLINE, and EMBASE). To capture all relevant articles, the following combination of terms and/or MESH headings were used: “uveitis”, “algorithms”, “artificial intelligence”, “decision support system, clinical”, and “Bayes Theorem”. An example search in PubMed was ((“uveitis” [MeSH Terms]) AND (“algorithms” [MeSH Terms] OR “artificial intelligence” [MeSH Terms] OR “decision support system, clinical” [MeSH Terms] OR “Bayes Theorem” [MeSH Terms])) (Table 1).

We included studies that reported the use of AI systems to determine any underlying etiology in patients with uveitis and/or as a tool to enhance the diagnosis or classification of uveitis. Meta-analyses, randomized controlled trials (RCTs), comparative and non-comparative clinical studies, systematic reviews, reviews, and consensus guidelines were potentially eligible. Single-case reports, animal studies, editorials, abstracts, and articles not written in English were excluded. The bibliographies of retrieved articles were searched for eligible studies. They were no limitations regarding the publication date.

### 2.2. Data Collection and Assessment

The retrieved abstracts were reviewed by two independent readers (one senior uveitis specialist and one senior internal medicine specialist), and, from these, articles were selected for full review. Uncertainties were resolved via discussion with another reviewer. Then, the full text of all the selected articles was read to determine the eligibility and collect data.

### 2.3. Risk of Bias Assessment and Outcome Measures

The risk of bias and applicability were assessed for primary diagnostic accuracy studies using the QUADAS-2 tool but not for secondary research (systematic reviews, reviews, and consensus guidelines) [13]. In the anticipated absence of many or any RCTs, we did not plan to undertake a meta-analysis or to pre-define any quantitative outcome measures, but rather, we aimed to synthesize and describe the available literature and identify areas for further research.

## 3. Results

### 3.1. Study Selection, Characteristics, and Risk of Bias

**Figure 1:**
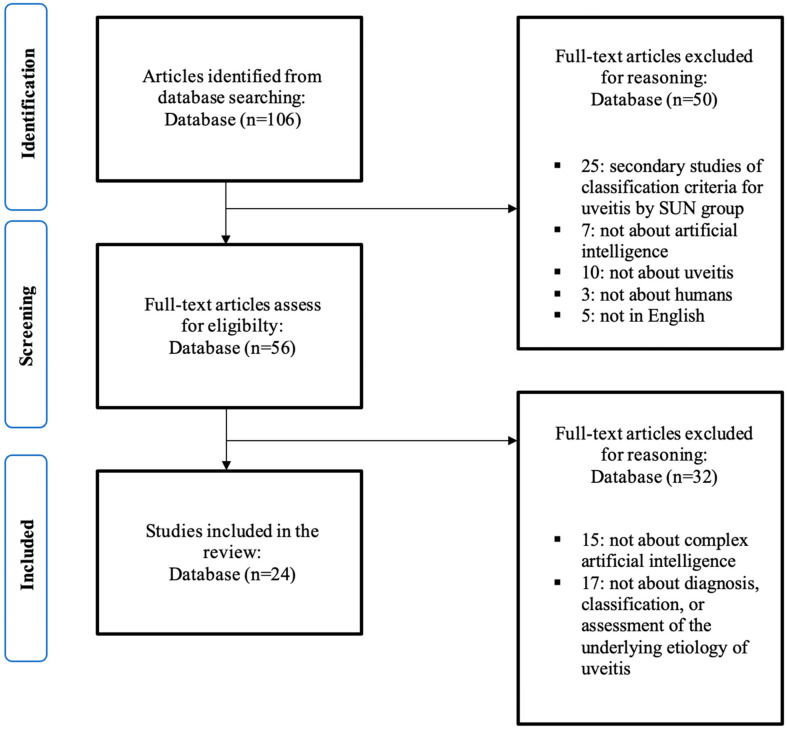
PRISMA flow diagram.

**Figure 2:**
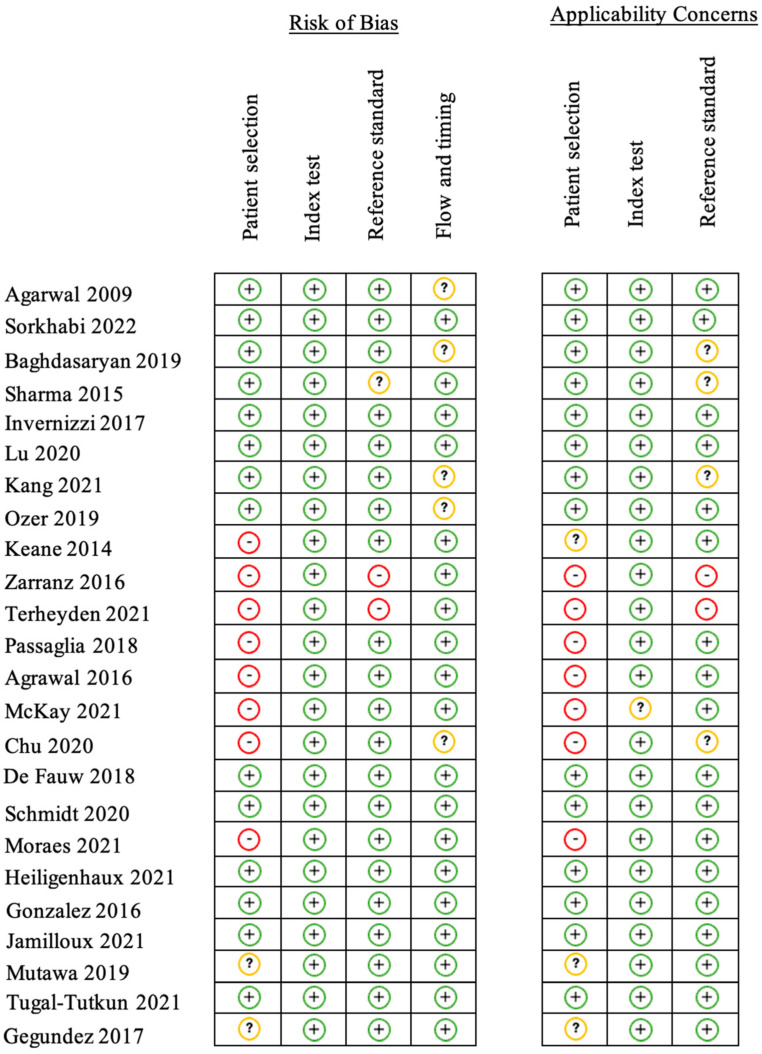
Risk of bias and applicability concerns table (QUADAS-2) [14]. Legend: Red (-) = high risk of bias; Yellow (?) = unknown risk of bias; Green (+) = low risk of bias [3,10,11,15,16,17,18,19,20,21,22,23,24,25,26,27,28,29,30,31,32,33,34,35].

We reviewed 106 abstracts, of which 56 articles were selected for full review (Figure 1). Of these, 24 were eligible for inclusion. These comprised 2 RCTs, 12 comparative clinical studies, 2 non-comparative clinical studies, 1 review, and 7 diagnostic accuracy studies. The articles detailing primary research included a total of 15,917 patients in 23 articles. The risk of bias was determined to be high in eight articles, low in nine articles, and unknown in seven articles. The risk of bias and applicability were determined for each study (Figure 2).

### 3.2. Diagnosis

#### 3.2.1. Anterior Segment

AI has been applied to medical imaging to assist with the diagnosis of uveitis. Researchers have demonstrated that AI-based analysis of anterior segment (AS) optical coherence tomography (OCT) images compared to a clinical examination with a slit lamp shows a significant and independent correlation with SUN classification for quantifying AS inflammation [15,16,17,18,19,20,21]. AS inflammation was detected by the identification of hyperreflective spots, which are then used as a representative for the AS. Agarwal et al. have shown a good correlation for hyperreflective spot count between automated and manual methods (Pearson coefficient for grade 1: 0.995, grade 2: 0.948, grade 3: 0.985, and grade 4: 0.893). There were no significant differences in mean values between the two methods except for grade 4. In this case, the automated method was more sensitive and detected a higher number of cells [15]. Similarly, Sorkhabi et al. have developed an automated AI-based method to quantify inflammation in the AS. They showed a significant correlation between clinical SUN grading and AI software-detected particle count (Spearman *p* = 0.7077) and particle density (Spearman *p* = 0.7035). AI-based image analysis of AS-OCT slides shows a significant and independent correlation with clinical SUN assessment [16]. Sharma et al. applied an automated algorithm to count the number of hyperreflective spots by AS spectral-domain OCT. The Spearman correlation coefficient was 0.967 between OCTs and clinical slit lamp evaluation. Interestingly, Ozer et al. suggested that the iris pigment optical density measured at the pupillary margin of spectral-domain OCT could be a marker of Fuch’s heterochromic uveitis [22].

#### 3.2.2. Vitreous Segment

The current gold standard for the quantification of vitreous inflammation, as recommended by the SUN working group, is clinical examination with an indirect ophthalmoscope, compared to a set of standard photographs. Several authors have suggested that macular OCT scans can offer greater reliability and accuracy by measuring the vitreous intensity [23,24,25]. Keane et al. provided a measurement of the vitreous signal intensity, which was then compared with that of the retinal pigment epithelium, generating an optical density ratio. These OCT-derived measurements showed a significant and positive correlation with clinical vitreous haze scores (r = 0.566) and a good degree of intergrader reproducibility (95% limit of agreement) [23]. Furthermore, Terheyden et al. suggested that 3 OCT b-scans might be sufficient to obtain a reliable automatic measurement of vitreous intensity [25]. Passaglia et al. designed an image-processing algorithm and compared computer scores against clinicians’ vitreous haze grading. Exact agreement between the algorithm and expert clinicians’ grades had a kappa value of 0.61 [26].

#### 3.2.3. Posterior Segment

Choroidal vascularity was used by Agrawal et al. to quantify choroidal inflammation [27]. They defined the choroidal vascularity index (CVI) as the proportion of the intraluminal subfoveal choroidal area to the total circumscribed subfoveal choroidal area in enhanced-depth imaging OCT. This was achieved by manually segmenting the choroid with custom software after processing it with ImageJ. They showed a 6.2% decrease in CVI between the time of uveitis diagnosis and 3-month follow-up, which was significantly higher than the 0.7% CVI decrease in control eyes. As such, CVI has potential as a novel tool for monitoring the clinical course of posterior uveitis, panuveitis, and choroidal disease.

McKay et al. proposed to identify and quantify inflammatory choriocapillaris lesions from automated swept-source OCT [28]. The algorithm demonstrated a high degree of agreement with human graders in the determination of lesion area, spatial overlap, and reproducibility. Chu et al. used similar methods to detect choriocapillaris flow attenuation, with significantly larger areas of flow attenuation in patients with posterior uveitis than uveitis with no posterior involvement [29].

Automated detection and quantification of macular edema already exist in neovascular age-related macular degeneration, diabetic retinopathy, and retinal vein occlusion [30,31,32]. Therefore, it could be applied to macular edema caused by uveitis, although intraocular inflammation could potentially obscure retinal structures [36]. Schmidt et al. and Moraes et al. applied a deep learning method for the localization and quantification of fluid in the retina using OCT scans, with promising results in neovascular age-related macular degeneration [32,36].

The potential utility of these advances is that AI may be able to minimize inter-observer variability and thereby facilitate more appropriate patient follow-up scheduling. It may also be able to assist, or conceivably even replace, clinicians’ classification of uveitis, and thereby triage severity. By identifying less severe cases, AI could provide ophthalmologists with more time to focus on sight-threatening diseases [37]. However, to date, none of these tools have been applied in clinical practice, and their impact on real-world diagnosis, treatment, and optimization of the clinical workflow needs to be evaluated by prospective, and ideally comparative, clinical studies.

### 3.3. Classification

AI has also been used in uveitis to process non-imaging clinical data. The SUN working group used AI to classify uveitis into 25 of the most common categories. First, they collected 4046 cases of uveitis from multiple uveitis centers on 5 continents with about 100–200 cases per entity, with the ground truth based on supermajority clinical agreement. Cases were split into a training set (~85% of the cases) and a validation set (~15% of the cases). Machine learning techniques through multinominal logistic regressions were then used within each uveitis subclass to classify patients with uveitis. The results with required and exclusion criteria showed a high degree of accuracy, ranging from 93.3% to 99.3% agreement, and appeared to perform well enough for use in clinical and translational research [3]. The final criteria of the 25 uveitic entities were later published.

Limitations include the lack of an external validation set, and in terms of clinical applicability, about half of the patients had a disease for which no SUN classification criteria existed [38]. Demographic data and bias assessment were not available in those papers, along with applied models and data that were not publicly available. There are, as of yet, no prospective or comparative studies detailing how the system performs in clinical practice.

### 3.4. Etiology

How one approaches the workup of patients with uveitis may determine how technology can assist in the process. An algorithmic approach to uncovering any underlying etiology may be the most efficient [39]. Such a systematic approach typically starts with history taking and demographics (age, gender, race, and socio-economic history). Secondly, the characteristics of ocular examination are considered (chronology, laterality, anatomical location, corneal changes, intraocular pressure, granulomatous changes, and the presence of synechiae, retinal vasculitis, papillary edema, macular edema, and focal or multifocal choroiditis and retinitis). Thirdly, the physical signs of extraocular disease can point to an underlying etiology. Given the potential benefits of a systematic approach, several AI algorithms have been designed to work with the resulting clinical data flow (Table 2).

In 2016, Gonzalez-Lopez et al. reported the results of a Bayesian belief network algorithm designed to help diagnose the cause of anterior uveitis [10]. The center node of the Bayesian network was the uveitis etiology (11 etiologies: idiopathic; ankylosing spondylitis; psoriatic arthritis; reactive arthritis; inflammatory bowel diseases; sarcoidosis; tuberculosis; Behçet disease [BD]; Posner–Schlossman syndrome; juvenile idiopathic arthritis; and Fuchs heterochromic cyclitis). Chance nodes included demographic data (gender), ophthalmic data (ocular symptoms and signs), extraocular data (systemic signs and symptoms), and laboratory findings, with a retrospective collection. In a dataset of 200 patients, the etiology (determined by the senior expert clinician) matched the first or second most likely diagnosis given by the algorithm in about 80.5% of cases. Interestingly, the algorithm was more useful for the exclusion of certain etiologies thanks to its high specificity (88.8% for sarcoidosis and 99.5% for Posner–Schlossman syndrome). The limitations of this algorithm were the absence of important conditions (syphilis, multiple sclerosis, and herpetic uveitis) and retrospective data collection.

In 2019, Mutawa et al. introduced a multilayered rule-based expert system to serve as a decision-making support system [33]. This medical expert system consisted of long-term memory, short-term memory, an inference engine, and a possible extra module for providing explanations. The system was tested on 61 cases, and the authors reported perfect agreement with human experts on each occasion. Indeed, the algorithm reported a match of 60.8% for all tested cases, overmatch (correct diagnosis among a list of probable diagnoses) in 39.2%, and no mismatch. However, in such cases, further investigations such as laboratory tests, would be necessary to exclude mimicking diseases. Furthermore, the sample size was limited, which may have affected the algorithm’s performance and generalizability.

Tugal-Tuktun et al. developed an algorithm for the diagnosis of uveitis associated with BD [34]. Firstly, they defined 10 ocular signs with a high diagnostic ratio for BD among 418 patients with uveitis (211 with BD and 207 with other uveitides). Secondly, they developed an algorithm using classification and regression tree analyses based on recursive partitioning analysis, applied to prospective data with a high-scoring tree re-evaluated for clinical relevance. The expert-guided diagnostic tree provided an area under the curve (AUC) of 0.92 (95% CI, 0.89–0.96), but this study was focused only on a single etiology.

Most recently, Jamilloux et al. used a Bayesian belief network on a dataset composed of 877 incident uveitis cases to identify the etiology (center node) [11]. Variables included age, gender, ethnicity, and the anatomic and clinical characteristics of the uveitis. To assess the algorithm performance, internal (by Monte Carlo cross-validation) and external validations were carried out. Network performance was quantified in terms of the proportion of patients correctly classified. Performance indicators were estimated for the most and the two most probable diagnoses. When the algorithm’s two most probable diagnoses were considered, they reported 80% agreement with the clinical diagnosis in the training dataset, confirmed at 85% in the independent validation set. Limitations of this study were collection from just one tertiary care center, which may affect generalizability to other centers and settings, and the risk of bias from retrospective data collection.

Two web-based diagnostic decision support systems (DDSSs) have been reported [35,40]. The first, Uveitis Doctor (Lara-Medina, Alcazar de San Juan, Spain), comprises more than 59 uveitis syndromes and an inference engine based on decision trees. The second, Uvemaster, contains a knowledge base of 88 uveitis syndromes acquired from the medical literature data, each comprising 76 clinical items. Each clinical sign was assigned a value of 0 to 100, depending on its prevalence for each specific uveitis syndrome. Then, the interference engine combines the filtering rules with the patient dataset in order to offer possible diagnoses with diagnostic performance indicators (sensitivity, specificity, or positive predictive value). Performance was assessed as the percentage of cases for which a specific diagnosis was obtained using the app. In a series of 228 patients, the diagnostic accuracy of the algorithm was 96.6%, and when the first diagnoses proposed by the app were considered, the sensitivity was 73.9% and the positive predictive value was 29.5%. Helpfully, it incorporates ophthalmic, demographic, and clinical data in the dataset, but there are relatively few cases reported relative to the number of potential causes.

These apps may improve the clinical management of uveitis by offering potential diagnoses and reducing the number of cases labeled as idiopathic uveitis. However, the knowledge base includes diagnoses with very high specificity tests and ophthalmic diagnoses that can usually be easily recognized. This could lead to an overestimation of the algorithm’s diagnostic performance, where results are extrapolated to other situations.

All these systems had acceptable or good performance, especially when the two most likely diagnoses were sought. However, the most common etiologies were considered in three of the systems, and it is not yet known how they will perform with rarer or more complex etiologies.

## 4. Discussion

In this systematic review, we summarize the contribution of AI in the diagnosis, classification, and assessment of the underlying etiology of uveitis. However, there are significant limitations in many studies, and the interpretation of results should be within these limitations. First, the design of the studies concerning uveitis diagnosis does not lead to high-level evidence recommendations, with only two randomized controlled trials. Before use in daily practice, we suggest that future studies test these AI-based image analyses for the diagnosis of uveitis in studies with higher methodological quality. Second, the validation of algorithms developed to assist clinicians in the assessment of the underlying etiology of uveitis was carried out on the test cohort in the majority of diagnostic accuracy studies. Only one used an external validation cohort, but both cohorts (test and validation) contained uveitis patients from the same country (France). This limits the international implementation of these algorithms, especially because of the heterogeneity of the uveitis causes between countries related to environmental influence.

Uveitis is an important ophthalmic disease worldwide, with multiple etiologies, a wide variation in its presentation, and a potentially complex differential diagnosis. This can lead to excessive testing and costs, delays in initiating the correct treatment, and a lack of clear information for patients. Any tools that enhance diagnostic accuracy and efficiency may therefore help optimize the management of patients with uveitis. Computer-aided systems may enhance diagnostic performance because of their greater capacity to process, compare, and summarize clinical data. They have shown potential in the diagnosis of uveitis, its classification, and in determining the underlying cause. McKay et al. have shown, using a Bayesian approach, that failure to consider all of the patient’s relevant characteristics reduces the ability to determine the underlying etiology and leads to overdiagnosis, greater cost to the healthcare system, and overtreatment [41]. Accordingly, existing algorithms may be improved by incorporating new demographic elements (e.g., ethnicity), additional ophthalmic variables (e.g., the existence of papilledema or focal or diffuse choroiditis), detailed clinical examination findings (e.g., oral aphthosis and arthralgias), and paraclinical data (e.g., laboratory tests and organ biopsy). Increasing the number of patients is also important. An AI system that incorporates these extra data and that is developed and tested in large patient numbers may facilitate individualized patient management by improving diagnostic accuracy (especially for non-expert clinicians). This will provide rapid and more targeted therapy as well as extend the reach of AI to more complex and rare diagnoses.

For this purpose, the evidence base needs to be improved, as, currently, there are few comparative studies and no relevant RCTs. To this aim, patient populations should be well characterized and described and the methodology should be accurately described, and the use of shared outcome measures (Table 3) will facilitate meta-analyses and a comparison between technologies.

## 5. Conclusions

AI algorithms show promising results to help clinicians in the diagnosis of uveitis, in its classification, and in determining the underlying cause. Future studies should aim to establish not only the diagnostic accuracy of new technologies but also that they enhance clinical and health economic outcomes in comparison with the standard of care. The inclusion of other etiologies of uveitis and more comprehensive data through collaborative research and data sharing seems essential to carry out well-designed, adequately powered RCTs.

## Figures and Tables

**Table 1:** MESH terms used for PUBMED research.

1	Uveitis [MeSH Terms] OR uveitides [MeSH Terms]
2	Algorithms [MeSH Terms]
3	Artificial intelligence [MeSH Terms] OR Computational Intelligence [MeSH Terms] OR Intelligence, Computational [MeSH Terms] OR Machine Intelligence [MeSH Terms] OR [MeSH Terms] OR Intelligence, Machine [MeSH Terms] OR Computer Reasoning [MeSH Terms] OR Reasoning, Computer [MeSH Terms] OR AI (Artificial Intelligence) [MeSH Terms] OR Computer Vision Systems [MeSH Terms] OR Computer Vision System [MeSH Terms] OR System, Computer Vision [MeSH Terms] OR Systems, Computer Vision [MeSH Terms] OR Vision System, Computer [MeSH Terms] OR Vision Systems, Computer [MeSH Terms] OR Knowledge Acquisition (Computer) [MeSH Terms] OR Acquisition, Knowledge (Computer) [MeSH Terms] OR Knowledge Representation (Computer) [MeSH Terms] OR Knowledge Representations (Computer) [MeSH Terms] OR Representation, Knowledge (Computer) [MeSH Terms]
4	Decision support systems, Clinical [MeSH Terms] OR Clinical Decision Support Systems [MeSH Terms] OR Clinical Decision Support System [MeSH Terms] OR Clinical Decision Support [MeSH Terms] OR Clinical Decision Supports [MeSH Terms] OR Decision Supports, Clinical [MeSH Terms] OR Support, Clinical Decision [MeSH Terms] OR Supports, Clinical Decision [MeSH Terms] OR Decision Support, Clinical [MeSH Terms]
5	Bayes Theorem [MeSH Terms] OR Theorem, Bayes [MeSH Terms] OR Bayesian Forecast [MeSH Terms] OR Forecast, Bayesian [MeSH Terms] OR Bayesian Prediction [MeSH Terms] OR Prediction, Bayesian [MeSH Terms] OR Bayesian Estimation [MeSH Terms] OR Estimation, Bayesian [MeSH Terms] OR Bayesian Analysis [MeSH Terms] OR Bayesian Approach [MeSH Terms] OR Approach, Bayesian [MeSH Terms] OR Approachs, Bayesian [MeSH Terms] OR Bayesian Approachs [MeSH Terms] OR Analysis, Bayesian [MeSH Terms] OR Bayesian Method [MeSH Terms] OR Method, Bayesian [MeSH Terms]

**Table 2:** Review of algorithm approach for etiological diagnosis of uveitis and their diagnostic performance.

Study	Methods	Study Factors	Etiology	Diagnostic Performance
Gonzalez et al., 2016 [10]	Bayesian belief networks Dataset: 200 cases (anterior uveitis)	Demographic (gender) Ophthalmic (ocular symptoms and signs) Clinical examination (systemic symptoms and signs) Laboratory tests	11	Sensitivity (most probable etiology): 64% Sensitivity (two most probable etiologies): 81%
Gegundez-Fernandez et al., 2017 (Uvemaster) [35]	Interference method with filtering rules Dataset: 88 cases (all uveitis type)	Demographic (age, gender, immunodeficiency, drugs, trauma or eye surgery, and endemic disease) Ophthalmic (anatomy, chronicity, laterality, granulomatous, vasculitis, papillitis, scleritis, and specific ocular involvement) Clinical examination (skin, mucosal, nervous system, articular, urinary, ear, nose, throat, and digestive and cardiovascular exam) Treatment (steroid response)	88	Sensitivity (most probable etiology): 74% Sensitivity (three most probable etiologies): 91%
Mutawa et al., 2019 [33]	Rule-based expert system (multilayer rule design) Dataset: 61 cases (case report, all uveitis type)	Ophthalmic (anatomy, chronicity, severity, laterality, granulomatous) Treatment (response to therapy)	53	Sensitivity (most probable etiology): 60% Sensitivity (four most probable etiologies): 100%
Jamilloux et al., 2021 [11]	Bayesian belief networks Dataset: 877 cases (all uveitis type)	Demographic (Age, sex, and ethnicity) Ophthalmic (Anatomy, laterality, chronicity, vasculitis, granulomatous, and ocular hypertension)	8	Sensitivity (most probable etiology): 54% Sensitivity (two most probable etiologies): 85%

**Table 3:** Suggested outcome metrics for reporting outcomes of diagnostic decision support systems in uveitis.

Type of Outcome	Examples of Indicators
Description of patient population	Number of participants Patients or case report Age Sex Ethnicity Co-morbidity
Setting, type of unit	Community, primary care, secondary care, tertiary care Mono/multicentric Nationality
Study factors	Demographic data Ocular examination data Extraocular examination data Laboratory tests results Response to treatment
Etiologies	Number of etiologies Rare etiologies or not? Etiologies with high specific tests?
Data collection method	Retrospective Prospective
Reference standard	Standard of diagnosis/etiology assessment: detailed description By whom: expert or not?
Computing methods	Type of algorithm used? Bayesian belief networks; random forest; interference method with filtering rules; rule-based expert system; tree analysis…
Outcome measures	Timepoint Sensitivity Specificity Positive predictive value Area under the receiver operating characteristic curve (AUROC)
Validation	Part of the testing cohort (separation before analysis in two parts) External cohort

## References

[1] Prete M., Dammacco R., Fatone M.C., Racanelli V. (2015). Autoimmune uveitis: Clinical, pathogenetic, and therapeutic features. Clin. Exp. Med..

[2] Dick A.D., Tundia N., Sorg R., Zhao C., Chao J., Joshi A., Skup M. (2015). Risk of Ocular Complications in Patients with Noninfectious Intermediate Uveitis, Posterior Uveitis, or Panuveitis. Ophthalmology.

[3] Heiligenhaus A., Rothaus K., Pleyer U. (2021). Development of classification criteria for uveitis by the standardization of uveitis nomenclature (SUN) working group. Ophthalmol. Z. Dtsch. Ophthalmol. Ges..

[4] Sève P., Cacoub P., Bodaghi B., Trad S., Sellam J., Bellocq D., Bielefeld P., Sène D., Kaplanski G., Monnet D. (2017). Uveitis: Diagnostic work-up. A literature review and recommendations from an expert committee. Autoimmun. Rev..

[5] Abad S., Sève P., Dhote R., Brézin A.P. (2009). Guidelines for the management of uveitis in internal medicine. Rev. Med. Interne.

[6] Bodaghi B., Cassoux N., Wechsler B., Hannouche D., Fardeau C., Papo T., Huong D.L., Piette J.C., LeHoang P. (2001). Chronic severe uveitis: Etiology and visual outcome in 927 patients from a single center. Medicine.

[7] de Parisot A., Kodjikian L., Errera M.-H., Sedira N., Heron E., Pérard L., Cornut P.-L., Schneider C., Rivière S., Ollé P. (2017). Randomized Controlled Trial Evaluating a Standardized Strategy for Uveitis Etiologic Diagnosis (ULISSE). Am. J. Ophthalmol..

[8] Seixas F.L., Zadrozny B., Laks J., Conci A., Muchaluat Saade D.C. (2014). A Bayesian network decision model for supporting the diagnosis of dementia, Alzheimer׳s disease and mild cognitive impairment. Comput. Biol. Med..

[9] Hamilton P.W., Anderson N., Bartels P.H., Thompson D. (1994). Expert system support using Bayesian belief networks in the diagnosis of fine needle aspiration biopsy specimens of the breast. J. Clin. Pathol..

[10] González-López J.J., García-Aparicio M., Sánchez-Ponce D., Muñoz-Sanz N., Fernandez-Ledo N., Beneyto P., Westcott M.C. (2016). Development and validation of a Bayesian network for the differential diagnosis of anterior uveitis. Eye.

[11] Jamilloux Y., Romain-Scelle N., Rabilloud M., Morel C., Kodjikian L., Maucort-Boulch D., Bielefeld P., Sève P. (2021). Development and Validation of a Bayesian Network for Supporting the Etiological Diagnosis of Uveitis. J. Clin. Med..

[12] Kras A., Celi L.A., Miller J.B. (2020). Accelerating ophthalmic AI research: The role of an open access data repository. Curr. Opin. Ophthalmol..

[13] Page M.J., McKenzie J.E., Bossuyt P.M., Boutron I., Hoffmann T.C., Mulrow C.D., Shamseer L., Tetzlaff J.M., Akl E.A., Brennan S.E. (2021). The PRISMA 2020 statement: An updated guideline for reporting systematic reviews. BMJ.

[14] Whiting P.F., Rutjes A.W., Westwood M.E., Mallett S., Deeks J.J., Reitsma J.B., Leeflang M.M., Sterne J.A., Bossuyt P.M. (2011). QUADAS-2: A revised tool for the quality assessment of diagnostic accuracy studies. Ann. Intern. Med..

[15] Agarwal A., Ashokkumar D., Jacob S., Agarwal A., Saravanan Y. (2009). High-speed Optical Coherence Tomography for Imaging Anterior Chamber Inflammatory Reaction in Uveitis: Clinical Correlation and Grading. Am. J. Ophthalmol..

[16] Sorkhabi M.A., Potapenko I.O., Ilginis T., Alberti M., Cabrerizo J. (2022). Assessment of Anterior Uveitis through Anterior-Segment Optical Coherence Tomography and Artificial Intelligence-Based Image Analyses. Transl. Vis. Sci. Technol..

[17] Baghdasaryan E., Tepelus T.C., Marion K.M., Huang J., Huang P., Sadda S.R., Lee O.L. (2018). Analysis of ocular inflammation in anterior chamber—Involving uveitis using swept-source anterior segment OCT. Int. Ophthalmol..

[18] Sharma S., Lowder C.Y., Vasanji A., Baynes K., Kaiser P.K., Srivastava S.K. (2015). Automated Analysis of Anterior Chamber Inflammation by Spectral-Domain Optical Coherence Tomography. Ophthalmology.

[19] Invernizzi A., Marchi S., Aldigeri R., Mastrofilippo V., Viscogliosi F., Soldani A., Adani C., Garoli E., Viola F., Fontana L. (2017). Objective Quantification of Anterior Chamber Inflammation: Measuring Cells and Flare by Anterior Segment Optical Coherence Tomography. Ophthalmology.

[20] Lu M., Wang X., Lei L., Deng Y., Yang T., Dai Y., Li Y., Gan X., Hu Y., Chen H. (2020). Quantitative Analysis of Anterior Chamber Inflammation Using the Novel CASIA2 Optical Coherence Tomography. Am. J. Ophthalmol..

[21] Kang T.S., Lee Y., Lee S., Kim K., Lee W.-S., Lee W., Kim J.H., Han Y.S. (2021). Development of fully automated anterior chamber cell analysis based on image software. Sci. Rep..

[22] Ozer M.D., Kebapci F., Batur M., Seven E., Tekin S. (2019). In vivo analysis and comparison of anterior segment structures of both eyes in unilateral Fuchs’ uveitis syndrome. Graefes Arch. Clin. Exp. Ophthalmol. Albrecht Von Graefes Arch. Klin. Exp. Ophthalmol..

[23] Keane P.A., Karampelas M., Sim D.A., Sadda S.R., Tufail A., Sen H.N., Nussenblatt R.B., Dick A.D., Lee R.W., Murray P.I. (2014). Objective measurement of vitreous inflammation using optical coherence tomography. Ophthalmology.

[24] Zarranz-Ventura J., Keane P.A., Sim D.A., Llorens V., Tufail A., Sadda S.R., Dick A.D., Lee R.W., Denniston A.K., Adan A. (2016). Evaluation of Objective Vitritis Grading Method Using Optical Coherence Tomography: Influence of Phakic Status and Previous Vitrectomy. Am. J. Ophthalmol..

[25] Terheyden J.H., Ometto G., Montesano G., Wintergerst M.W.M., Langner M., Liu X., Keane P.A., Crabb D.P., Denniston A.K., Finger R.P. (2021). Automated quantification of posterior vitreous inflammation: Optical coherence tomography scan number requirements. Sci. Rep..

[26] Passaglia C.L., Arvaneh T., Greenberg E., Richards D., Madow B. (2018). Automated Method of Grading Vitreous Haze in Patients with Uveitis for Clinical Trials. Transl. Vis. Sci. Technol..

[27] Agrawal R., Salman M., Tan K.A., Karampelas M., Sim D.A., Keane P.A., Pavesio C. (2016). Choroidal Vascularity Index (CVI)—A Novel Optical Coherence Tomography Parameter for Monitoring Patients with Panuveitis?. PLoS ONE.

[28] McKay K.M., Chu Z., Kim J.-B., Legocki A., Zhou X., Tian M., Munk M.R., Wang R.K., Pepple K.L. (2021). Automated Quantification of Choriocapillaris Lesion Area in Patients with Posterior Uveitis. Am. J. Ophthalmol..

[29] Chu Z., Weinstein J.E., Wang R.K., Pepple K.L. (2020). Quantitative Analysis of the Choriocapillaris in Uveitis Using En Face Swept-Source Optical Coherence Tomography Angiography. Am. J. Ophthalmol..

[30] De Fauw J., Ledsam J.R., Romera-Paredes B., Nikolov S., Tomasev N., Blackwell S., Askham H., Glorot X., O’Donoghue B., Visentin D. (2018). Clinically applicable deep learning for diagnosis and referral in retinal disease. Nat. Med..

[31] Schmidt-Erfurth U., Vogl W.-D., Jampol L.M., Bogunović H. (2020). Application of Automated Quantification of Fluid Volumes to Anti–VEGF Therapy of Neovascular Age-Related Macular Degeneration. Ophthalmology.

[32] Moraes G., Fu D.J., Wilson M., Khalid H., Wagner S.K., Korot E., Ferraz D., Faes L., Kelly C.J., Spitz T. (2020). Quantitative Analysis of OCT for Neovascular Age-Related Macular Degeneration Using Deep Learning. Ophthalmology.

[33] Mutawa A.M., Alzuwawi M.A. (2019). Multilayered rule-based expert system for diagnosing uveitis. Artif. Intell. Med..

[34] Tugal-Tutkun I., Onal S., Stanford M., Akman M., Twisk J.W., Boers M., Oray M., Özdal P., Kadayifcilar S., Amer R. (2020). An Algorithm for the Diagnosis of Behçet Disease Uveitis in Adults. Ocul. Immunol. Inflamm..

[35] Gegundez-Fernandez J.A., Fernández-Vigo J.I., Diaz-Valle D., Mendez-Fernandez R., Cuiña-Sardiña R., Santos-Bueso E., Benitez-Del-Castillo J.M. (2017). Uvemaster: A Mobile App-Based Decision Support System for the Differential Diagnosis of Uveitis. Investig. Opthalmol. Vis. Sci..

[36] Abellanas M., Elena M.J., A Keane P., Balaskas K., Grewal D.S., Carreño E. (2022). Artificial Intelligence and Imaging Processing in Optical Coherence Tomography and Digital Images in Uveitis. Ocul. Immunol. Inflamm..

[37] Ahuja A.S., Wagner I.V., Dorairaj S., Checo L., Hulzen R.T. (2022). Artificial intelligence in ophthalmology: A multidisciplinary approach. Integr. Med. Res..

[38] Mudie L.I., Reddy A.K., Patnaik J.L., Pecen P., Kim E., Cole K., Palestine A.G. (2022). Evaluation of the SUN Classification Criteria for Uveitides in an Academic Uveitis Practice. Am. J. Ophthalmol..

[39] Rathinam S.R., Babu M. (2013). Algorithmic approach in the diagnosis of uveitis. Indian J. Ophthalmol..

[40] de la Torre-Díez I., Martínez-Pérez B., López-Coronado M., Díaz J.R., López M.M. (2015). Decision support systems and applications in ophthalmology: Literature and commercial review focused on mobile apps. J. Med. Syst..

[41] McKay K.M., Lim L.L., Van Gelder R.N. (2021). Rational laboratory testing in uveitis: A Bayesian analysis. Surv. Ophthalmol..

